# Takotsubo Cardiomyopathy in a Patient With Multiple Autoimmune Disorders and Hyperthyroidism

**DOI:** 10.5812/cardiovascmed.10023

**Published:** 2013-07-31

**Authors:** Murat Ugurlucan, Yilmaz Zorman, Gursel Ates, Ahmet H. Arslan, Yahya Yildiz, Aysegul Karahan Zor, Sertac Cicek

**Affiliations:** 1 Anadolu Medical Center Hospital, Cardiovascular Surgery Clinic, Istanbul, Turkey; 2 University Hospitals Geneva, Division of Cardiovascular Surgery, Geneva, Switzerland

**Keywords:** Takotsubo Cardiomyopathy, Autoimmue Disease, Ventricular Dysfunction, Left, Hyperthyroidism

## Abstract

Takotsubo cardiomypathy is a very rare cardiovascular syndrome leading to myocardial infarction and left ventricular dysfunction in the absence of a detectable coronary artery lesion. It is accepted as reversible left ventricular asynergy occuring typically after an intrinsic adrenergic hyperstimulation. In this report we present Takotsubo cardiomyopathy in a 75-year-old patient with multiple autoimmune disorders.

## 1. Introduction

Takotsubo cardiomyopathy is a rare disorder leading to left ventricular dysfunction in the absence of a critical coronary artery lesion (1-5). The disorder may also be named as neurologic myocardial stunning, stress induced cardiomyopathy, ampulla cardiomyopathy, transient left ventricular ballooning or broken heart syndrome (6). It is speculated that it usually resulted from transient coronary vasospasm and micorvascular dysfunction secondary to an adrenergic stimulus which increases the circulating levels of catecholamines (7). In the literature, the most frequent stimuslus leading to the condition has been reported to be hyperthyroidism or thyrotoxicosis (8-15). In this report, we present Takotsubo cardiomyopaty in a 75-year-old female patient with multiple autoimmune diseases.

## 2. Case Report

The patient was a 75-year-old woman presented to the emergency clinic with acute onset dyspnea and chest pain. Her history revealed medically controlled hypertension, Hashimoto thyroiditis with hyperthyroidism, pernicious anemia, and rheumatoid arthritis. Chest X-ray was unremarkable. Electrocardiography indicated ischemia at the left anterior descending coronary artery (LAD) territory. Blood chemistry revealed increased Troponin-I (3.450 ng/ml; N: < 0.02 ng/ml), creatinine kinase-MB (51.60 ng/mL; N: 1.00-4.99ng/ml). She was diagnosed as acute coronary syndrome and taken to the catheterization lab. 

**Figure 1.**
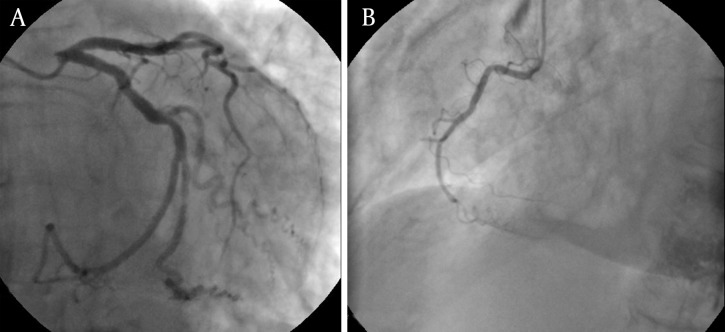
Coronary Angiography did not Indicate any Significant Stenosis, A: Left coronary system, B: Right coronary system

**Figure 2.**
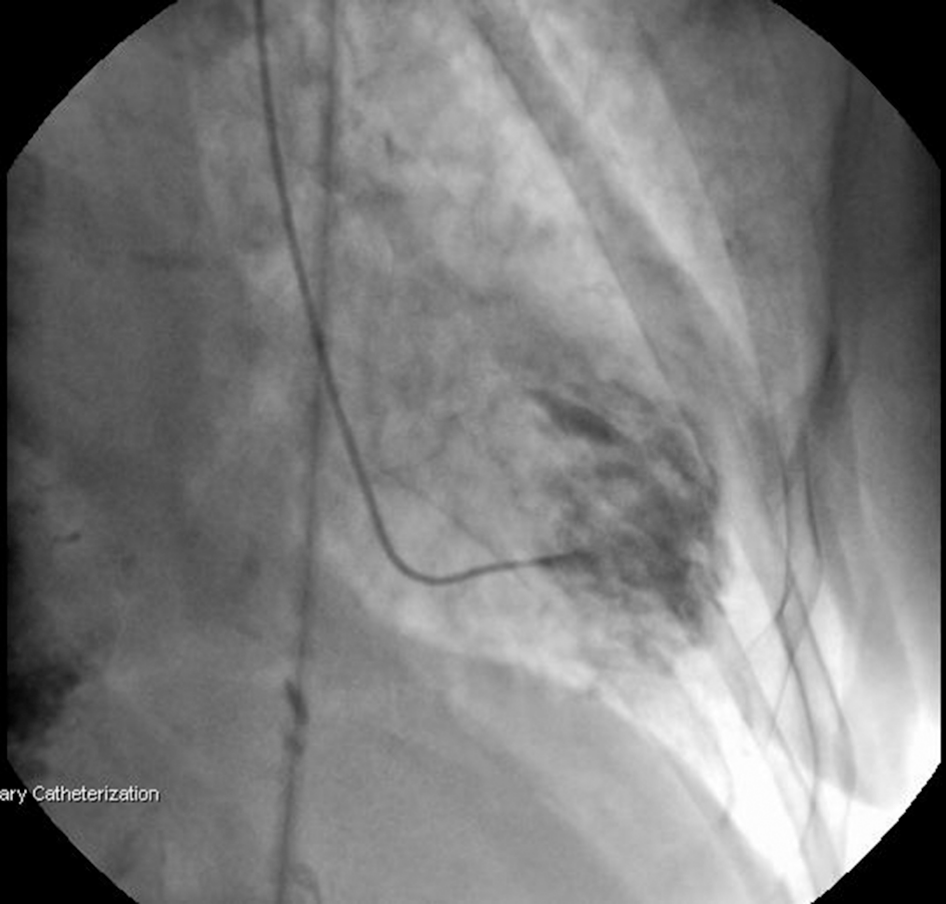
Left Ventriculography Showing Anteroapical and Lateral Dyskinesia

**Figure 3.**
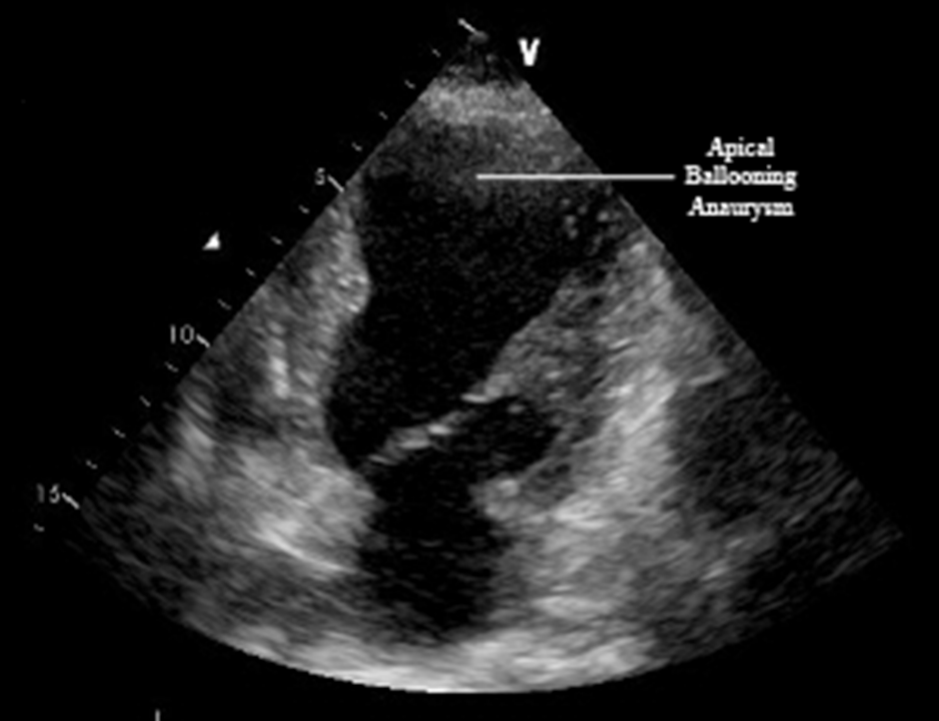
Apical Ballooning (Aneurysm Formation)

Coronary angiography was performed through radial artery. No significant stenosis was detected in any of the coronary arteries; however, there was slow flow and increased thrombus load in the LAD (Figure 1 A and B). Ventriculography showed anteroapical and lateral dyskinesia (Figure 2). Ventricular fibrillation developed during fractional flow reserve measurement of the LAD and the patient was defibrillated. The procedure was terminated. The patient was taken to the intensive care unit (ICU) and tirofibran, fractionated heparin (0,6ml b.i.d), aspirin (300mg/day), clopidogrel (75mg/day) and diuretics (spironolactone 25mg/day and furosemide 40mg b.i.d) were started. Further blood tests indicated anemia with hemoglobin level of 10,4gr/dl, increased thyroid hormon levels with suppressed thyroid stimulating hormon [fT4: 4,30 ng/dL (N: 0,93-1,70 ng/dL), fT3: 14,37 pg/mL (N: 1,80-4,60 pg/dL), TSH: 0,074 uIU/mL (N:0,270-4,200 uIU/mL)], increased D-Dimer (1.58 meq/Ml, N:0,06-0,7), CRP (7.80 mg/L, N: 0,01-5,00), Pro-BNP (2350 pg/mL; N: < 0.125 pg/mL) and liver enzymes (AST, 101 U/L; N: 5,00-31,00U/L; ALT: 48 U/L; N:3,0-40,00); otherwise normal. Due to the hyperthyroid status, propylthyouraci dose was increased to 100mg t.i.d. Echocardiography revealed segmentary wall motion abnormality with septal akinesia, anteroapical dyskinesia, moderate mitral valve insufficiency, increased pulmonary artery pressure (70/30mmHg with a mean of 50mmHg) and ejection fraction of 30%. Interestingly, there was apical ballooning (Figure 3). 

Her condition was stabilized and she was taken to the ward on the second day; however, she was re-admitted to the ICU due to acute pulmonary edema on the third day. Another single dose of tirofibran was infused and 5mcg/kg/minute of dobutamine was started. Her condition improved rapidly and control echocardiography showed better myocardial wall motions, increased ejection to 45% and decreased pulmonary mean pressure to 35mmHg on the 5th day. Rest of her stay was uneventful and she was discharged home after 4 days. She has been followed at the outpatient clinic regularly and the echocardiography on the 5th week indicated relatively normalized myocardial functions such as attenuated pulmonary pressure, trivial mitral regurgitation and 55% ejection fraction.

## 3. Discussion

Takotsubo cardiomyopathy or so called apical ballooning cardiomyopathy usually occurs following emotional or physical stres. It is characterized by acute and reversible left ventricular systolic dysfunction in the presence of typical electrocardiographic changes despite a detectable critical coronary lesion (1-6). The disease was first reported in 1991 by Dote et al. (16) in Japan. The shape of the heart, due to apical aneurysm in the early course of the disease resembles the ‘octopus trap’ used in Japan by fishermen which is ‘tako-tsubo’ in Japanese and the name of the disease comes from this entity (17). The pathophysiologic mechanism leading to the condition is unclear. Definetely there is myocardial ischemic insult which can be detected by biochemical markers such as increased troponin and creatinine kinase-MB levels. Moreover, electrocardiographic changes especially on septal and anterolateral leads are prominent. Coronary angiography does not show a considerable lesion in any of the vessels despite the symptoms of acute coronary syndrome. On the other hand, there should be at least ischemia at the mircocirculatory level.

A few machanisms have been proposed for Takotsubo cardiomyopathy. Since the disease is preceded by an emotinoal stimulus, the widely accepted underlying pathophysiology seems to be the circulating high levels of catecholamines and resulting in myocardial microvascular alterations. Takotsubo cardiomyopathy has frequently been reported in patients with increased thyroid hormones and it is well known that thyrotoxicosis is associated with augmented cathecholamine response (8-15). On the other hand, Nef et al. (18) state that the cathecholamines bind to the beta-2 adrenoreceptors which causes the activation of inhibitory protein G, so called the stimulus trafficking phenomenon, through cyclicAMP and it leads to negative inotropic effect as well as anti-apoptotis through PI3K-AKT signalling pathway in case of Takotsubo cardiomyopathy.

In the literature, association of Takotsubo cardiomyopathy with autoimmune disorders have also been reported. Our patient had both phenomea, i.e, both increased levels of circulating thyroid hormons secondary to Hashimoto thyroiditis and rheumatoid arthritis as well as pernicious anemia. Cakir et al. (15) reports that not only the increased thyroid hormones lead to Takotsubo cardiomyopathy but also it may occur as a result of complication of the autoimmunity of the underlying thyroid disease. Other than the cathecholamine and autoimmunity theories, acute coronary occlusion followed by rapid recanalization of the lumen and coronary vasospasm have also been speculated (19). 

The disease is usually seen in elderly postmenopausal women. Patients may present to the clinic with wide range of symptoms related to acute coronary syndrome. Except the angiographic findings, Takotsubo cardiomyopathy should be accounted among acute coronary syndromes. Hence, all the consequences of acute coronary syndromes, to the worst, ventricular fibrillation or death may ensue during the course of the disease. In the early phase of the disease left ventricular functions are compromised due to myocardial injury and typical apicolateral aneurysm formation. However, interestingly the findings have been reported to resolve in 2-9 weeks. In our patient, myocardial functions followed with echocardiography showed the healed heart in 5 weeks.

There is Kounis syndrome in the differential diagnosis of Takotsubo cardiomyopathy. Alhough both may be accounted among the acute coronary syndromes in the absence of coronary lesions, in case of Kounis syndrome an allergic reaction usually secondary to the administration of a medical agent is present and left ventricular aneurysm formation does not occur (20, 21). In conclusion, to the best of our knowledge, this is the first report in the literature of Takotsubo cardiomyopathy in a patient with both thyrotoxicosis and autoimmune disorders.
